# Recurrent Pleural Effusion as the Initial Presentation of T-lineage Lymphoblastic Malignancy in a Child: A Diagnostic Challenge

**DOI:** 10.7759/cureus.116811

**Published:** 2026-09-23

**Authors:** Hadi Fakih, Fatima Fakih

**Affiliations:** 1 Pediatrics, Sheikh Ragheb Harb University Hospital, Nabatieh, LBN; 2 Pediatrics, Faculty of Medical Sciences, Lebanese University, Beirut, LBN; 3 Neonatology, Faculty of Medicine, Lebanese University, Beirut, LBN

**Keywords:** diagnostic challenge, flow cytometry, pediatric malignancy, pleural effusion, t-cell lymphoblastic lymphoma

## Abstract

Pleural effusion in children is most frequently associated with infectious diseases, particularly parapneumonic effusion. Recurrent or unexplained pleural effusion, however, should prompt consideration of less common etiologies, including autoimmune disease, tuberculosis, chylothorax, cardiac and renal disease, and malignancy. We report the case of a 10-year-old boy with recurrent large right-sided pleural effusions requiring repeated drainage of more than 1,500 mL. An extensive diagnostic evaluation for rheumatologic and infectious causes was unrevealing. During the current admission, the patient had anorexia, weight loss, axillary lymphadenopathy, leukocytosis, and mild thrombocytopenia, without reported peripheral blood blasts. Chest computed tomography demonstrated a large right pleural collection, bilateral axillary lymphadenopathy, and prominent thymic tissue without a definite bulky anterior mediastinal mass. Pleural-fluid flow cytometry demonstrated an abnormal immature T-lineage lymphoblast population comprising approximately 99% of analyzed cells. Pleural biopsy showed tissue infiltration by atypical lymphoid cells morphologically resembling non-Hodgkin lymphoma. Subsequent bone marrow examination, performed after three days of methylprednisolone therapy, demonstrated diffuse blast infiltration with an immature T-cell immunophenotype and was diagnostic of T-cell acute lymphoblastic leukemia. Lymph-node biopsy showed lymphoproliferative disease, while lumbar puncture was normal. Pleural-fluid tuberculosis PCR and acid-fast bacilli testing were negative. This case emphasizes that recurrent pleural effusion may be an initial and predominant manifestation of T-cell acute lymphoblastic leukemia, even in the absence of peripheral blood blasts or a typical mediastinal mass. Early pleural-fluid flow cytometry may facilitate diagnosis and prevent diagnostic delay.

## Introduction

Pleural effusion is relatively uncommon in children compared with adults and has a different etiological spectrum. Infectious diseases, particularly parapneumonic effusion and empyema, account for a substantial proportion of pediatric cases. Other causes include tuberculosis, chylothorax, cardiac disease, renal and systemic disorders, autoimmune and inflammatory diseases, gastrointestinal pathology, and malignancy.

Although malignant pleural effusion is uncommon in children, it represents an important diagnostic consideration when an effusion is recurrent, unexplained, rapidly reaccumulating, or associated with lymphadenopathy, constitutional symptoms, or abnormal hematologic findings. Pediatric malignant pleural effusions may result from direct pleural infiltration, lymphatic obstruction, mediastinal or nodal disease, or dissemination from an underlying malignancy. Published pediatric cases have described both lymphoma and acute leukemia presenting with pleural effusion [1,2].

T-cell lymphoblastic neoplasms comprise a spectrum that includes T-cell lymphoblastic lymphoma (T-LBL) and T-cell acute lymphoblastic leukemia (T-ALL). These entities share a common immature T-cell phenotype and substantial biological overlap. Their clinical classification is principally influenced by the extent of bone marrow involvement, with T-ALL defined by substantial marrow involvement, conventionally ≥25% lymphoblasts, whereas T-LBL has absent or limited marrow involvement [3,4].

T-LBL has a strong predilection for the mediastinum and commonly occurs in children and adolescents. Pleural and pericardial effusions may accompany thoracic disease. However, the absence of a typical bulky anterior mediastinal mass does not exclude a T-lineage lymphoblastic malignancy [3,5].

The present case illustrates a diagnostic challenge in which recurrent large pleural effusion preceded recognition of T-ALL. The case emphasizes the importance of maintaining a broad differential diagnosis, recognizing that peripheral blood findings may be nonspecific or lack circulating blasts, and considering early flow cytometric examination of pleural fluid when a lymphoid malignancy is suspected.

## Case presentation

A 10-year-old boy was evaluated for recurrent pleural effusion. The patient had been admitted twice previously to another hospital for right-sided pleural effusion requiring chest tube insertion and drainage of more than 1,500 cc of fluid each time. During the month preceding his current admission, the patient had experienced anorexia, weight loss of 1 kg, and a sensation of heat. An echocardiogram performed during the prior evaluation was normal.

Prior to the current admission, an extensive investigation had been undertaken to identify infectious, autoimmune, and other systemic causes at an outside hospital. The workup is presented in Tables 1, 2.

**Table 1:** Summary of laboratory and immunological investigations performed at an external institution prior to the current admission. c-ANCA: cytoplasmic antineutrophil cytoplasmic antibody; p-ANCA: perinuclear antineutrophil cytoplasmic antibodies; PPD: purified protein derivative; TB: tuberculosis

Investigation	Patient result	Normal reference value	Interpretation
Rheumatoid factor (RF)	6.84 IU/mL	<14 IU/mL	Negative
Anti-cyclic citrullinated peptide (anti-CCP)	<1 U/mL	<5 U/mL	Negative
Antinuclear antibodies (ANA)	<1/100	Titer < 1/100	Negative
c-ANCA	Negative	Negative	Negative
p-ANCA	Negative	Negative	Negative
Immunoglobulin A (IgA)	83 mg/dL	53-204 mg/dL	Within normal range
Immunoglobulin G (IgG)	683 mg/dL	698-1,560 mg/dL	Mildly decreased
Immunoglobulin M (IgM)	125 mg/dL	31-179 mg/dL	Within normal range
PPD/tuberculin skin test	Negative	Negative	No evidence of TB infection by PPD
C-reactive protein (CRP)	0.3 mg/L	<10 mg/L	Normal
Erythrocyte sedimentation rate (ESR)	4 mm/hr	<15 mm/hr	Normal

**Table 2:** Complete blood count performed at the outside hospital. MCV: mean corpuscular volume; MCH: mean corpuscular hemoglobin; MCHC: mean corpuscular hemoglobin concentration; RDW-CV: red cell distribution width-coefficient of variation; WBC: white blood cell

Test	Result	Normal range
Red blood cells	5.40 × 10⁶/µL	4-5
Hemoglobin	15.4 g/dL	11-14
Hematocrit	44.8%	32-40
MCV	83.0 fL	80-96
MCH	28.5 pg	26-32
MCHC	34.4 g/dL	32-36
RDW-CV	13.5%	11-15
WBC	5.93 × 10³/µL	4.5-13.5
Neutrophils	33.0%	40-65
Lymphocytes	55.0%	20-45
Monocytes	7.3%	1-9
Eosinophils	4.4%	0-4
Basophils	0.3%	0-2
Platelets	232 × 10³/µL	150-450

Blood smear revealed normocytic normochromic red blood cells (RBCs) with no evidence of hemolysis and few atypical lymphocytes. Platelets showed few giant platelets. Uric acid was 6.0 mg/dL (3.4-7.0 mg/dL), and lactate dehydrogenase (LDH) was 240 U/L (120-300 U/L). Despite this extensive evaluation and repeated pleural drainage, the effusion recurred.

On the current admission, the patient was not in respiratory distress and was breathing comfortably without supplemental oxygen. Auscultation revealed decreased air entry at the basal right lung, but no cough was present. Right axillary enlarged lymphadenopathies were identified; these were painful and mobile. No hepatosplenomegaly was detected, and no skin rashes were observed. Laboratory findings showed an elevated white blood cell count of 32.55 × 10³/µL (reference: 5.0-13.0 × 10³/µL), with differential showing neutrophils 47.4%, lymphocytes 42.5%, monocytes 8.9%, eosinophils 0.9%, and basophils 0.3%. Immature granulocytes were present at 7.2%. The platelet count was low at 140 × 10³/µL (reference: 150-400 × 10³/µL). Hemoglobin was 13.4 g/dL (reference: 9.5-15 g/dL), and hematocrit was 37.5% (reference: 30%-44%).

The leukocytosis and mild thrombocytopenia were nonspecific findings and, in the absence of reported peripheral blasts, could not by themselves establish a diagnosis of leukemia or lymphoma. However, when considered together with the recurrent unexplained pleural effusion (Figure 1), lymphadenopathy, constitutional symptoms, and thoracic imaging abnormalities (Figure 2), they prompted further evaluation for an underlying hematologic or lymphoproliferative disorder.

**Figure 1:**
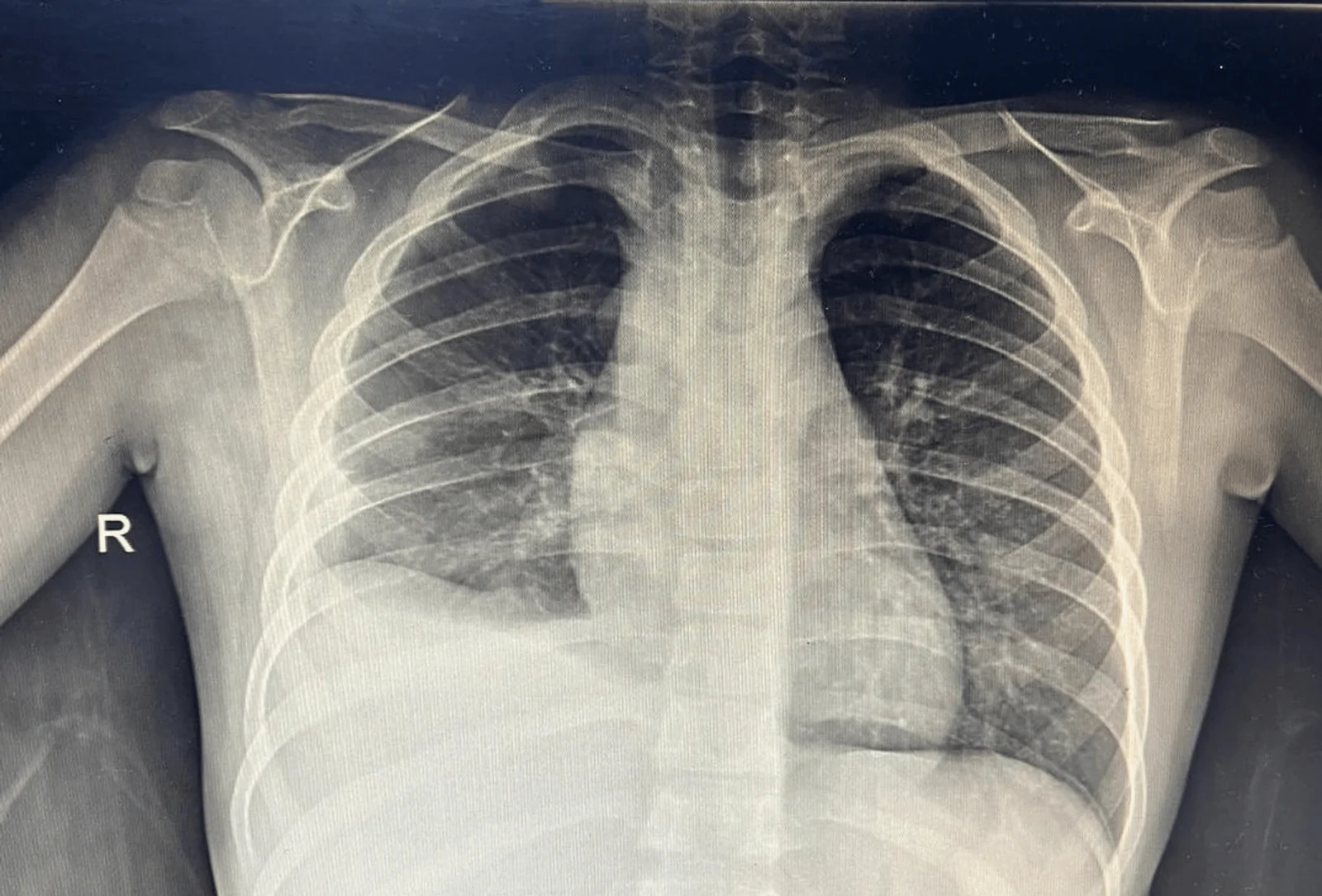
Chest radiograph AP view demonstrating the right pleural effusion.

**Figure 2:**
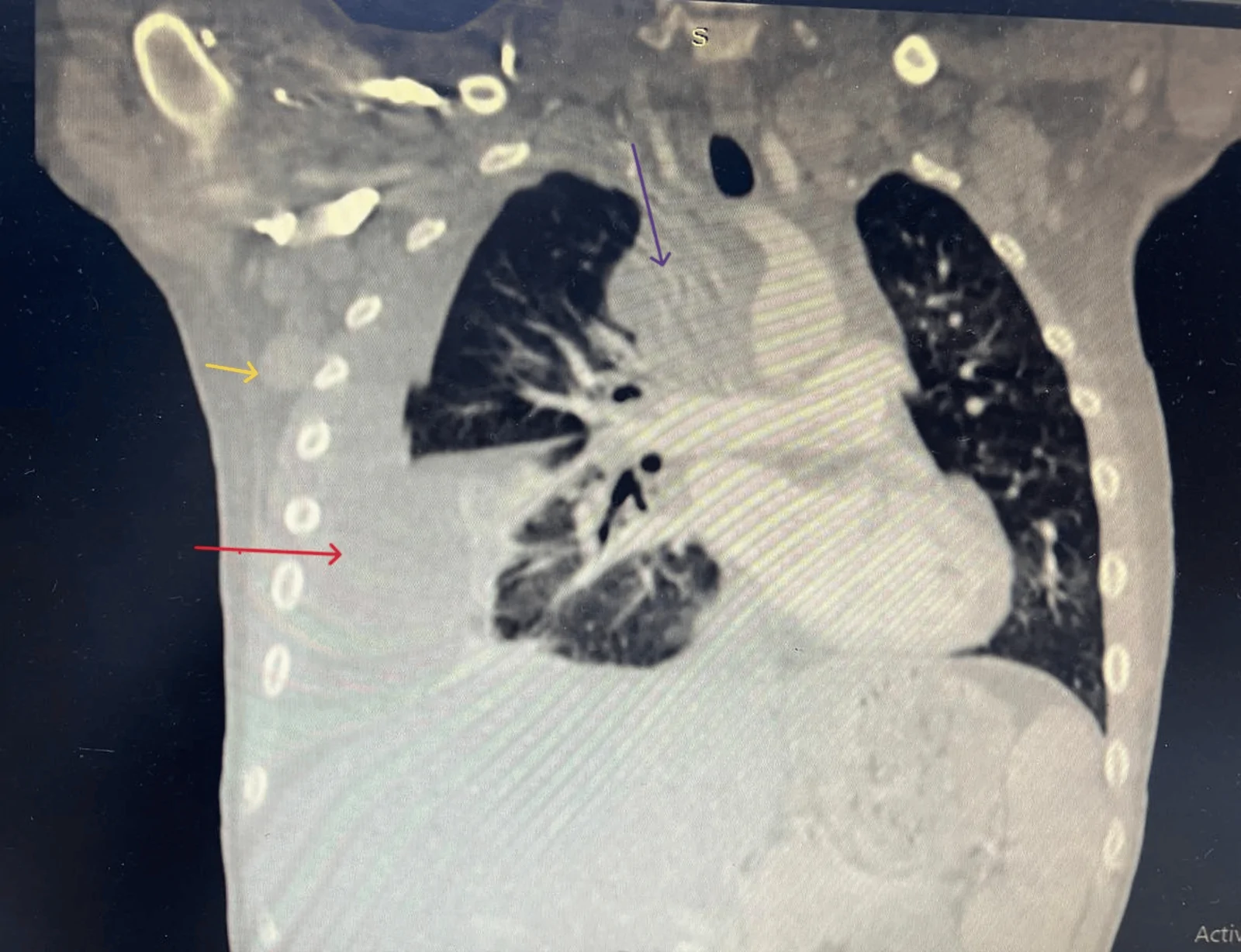
Contrast-enhanced computed tomography (CT) of the chest demonstrating the thoracic/pleural and mediastinal abnormalities. The CT chest demonstrated a 15 × 6 cm localized fluid area in the right lung (red arrow) with ipsilateral multiple lymphadenopathies, the largest measuring 3 cm at the right subaxillary region (yellow arrow). Thymus tissue extension to the anterior part of the heart was noted (violet arrow), prompting consideration of thymoma versus lymphoma. The left subaxillary region revealed multiple 13 mm lymph nodes. The superior mediastinum, paratracheal and aorto-pulmonary window, carina, hili, and lung parenchyma appeared normal.

A right-sided chest tube was inserted, and approximately 1,600 mL of serosanguinous pleural fluid was drained. Pleural-fluid analysis demonstrated a white blood cell count of 42,071 cells/µL, with mononuclear cells comprising 99.8% and polymorphonuclear cells 0.2%. The RBC count was 21 × 10³/µL. Pleural-fluid protein was 44.6 g/dL, and LDH was 547 U/L. Pleural-fluid tuberculosis PCR and acid-fast bacilli testing were negative.

Because of the marked mononuclear predominance, flow cytometric analysis of the pleural fluid was performed. It demonstrated an abnormal immature T-lineage lymphoblast population representing approximately 99% of analyzed cells. The abnormal population expressed CD1a, CD2, partial surface CD3, bright cytoplasmic CD3, CD4, CD5, CD7, CD38, CD45, and dim cytoplasmic TdT. The population was negative for the tested B-cell and myeloid markers. The laboratory interpretation was consistent with T-lymphoblastic leukemia/lymphoma of cortical/thymic phenotype [5]. A pleural biopsy was also performed. Macroscopic examination demonstrated four fragments of pleural tissue measuring approximately 1-4 cm. Histopathological examination showed fibro-fatty and skeletal muscle tissues heavily infiltrated by atypical small-to-medium-sized lymphoid cells, with numerous macrophages. The morphological appearance resembled a non-Hodgkin lymphoma.

Given the pleural-fluid flow cytometry and tissue findings, an underlying T-lineage lymphoblastic malignancy was strongly suspected. Methylprednisolone (Solu-Medrol) 40 mg intravenously once daily was initiated because of the clinical suspicion of a lymphoproliferative malignancy and concern regarding tumor lysis during further diagnostic management. Allopurinol 300 mg once daily, sodium bicarbonate 80 mEq/day for urine alkalinization, furosemide 20 mg twice daily, and intravenous hydration were also administered. Urine output, fluid balance, serum chemistry, and complete blood counts were monitored closely [6]. The patient remained under close clinical observation during this period.

Bone marrow aspiration/biopsy and lymph-node biopsy were subsequently performed after three days of Solu-Medrol therapy. Bone marrow examination showed a highly cellular marrow, approximately 100% cellular, diffusely infiltrated by mononuclear cells compatible with blasts, with rare megakaryocytes. Bone marrow flow cytometry demonstrated blasts expressing CD1a, CD2, cytoplasmic CD3, partial surface CD3, CD4, dim CD5, CD7, CD8, dim CD45, and TdT. The blasts were negative for CD19, CD34, CD79a, CD117, HLA-DR, and myeloperoxidase.

The final bone marrow diagnosis was T-ALL. The lymph-node biopsy demonstrated lymphoproliferative disease. Lumbar puncture was normal. The subsequent marrow findings established that the patient had T-ALL rather than T-LBL, because of extensive bone marrow involvement. The pleural and lymph-node findings represented extramedullary involvement by the same T-lineage lymphoblastic process.

The patient was classified by the treating pediatric hematology-oncology team as having low-to-intermediate-risk T-ALL. At the time of preparation of this report, disease-specific chemotherapy had not yet been initiated. The principal objective of this report is therefore to emphasize the diagnostic challenge and the importance of considering lymphoblastic malignancy in children with recurrent unexplained pleural effusion.

## Discussion

Recurrent pleural effusion in a child requires systematic reassessment of the differential diagnosis (Table 3) when the initial evaluation does not establish an etiology. Infectious causes, particularly parapneumonic effusion and empyema, remain important considerations. However, persistent or recurrent effusion should prompt consideration of alternative infectious, inflammatory, lymphatic, systemic, and malignant causes [6].

**Table 3:** Differential diagnosis of recurrent pleural effusion in children. SLE: systemic lupus erythematosus

Category	Important causes
Infectious	Parapneumonic effusion/empyema, tuberculosis, fungal infection
Malignant	Lymphoma, leukemia, metastatic malignancy, primary pleural tumors
Chylous	Thoracic duct injury, congenital lymphatic disorders, lymphoma
Cardiac	Congestive heart failure, congenital heart disease
Renal/systemic	Nephrotic syndrome, renal failure, hypoalbuminemia
Autoimmune/inflammatory	SLE, juvenile idiopathic arthritis, other connective-tissue diseases
Gastrointestinal	Pancreatitis, esophageal perforation, subdiaphragmatic inflammation
Other	Pulmonary embolism, lymphatic abnormalities

In the present case, several features ultimately supported a malignant rather than an infectious or autoimmune process. These included the repeated large-volume recurrence of the effusion, constitutional symptoms, axillary lymphadenopathy, a new leukocytosis with mild thrombocytopenia, thoracic imaging abnormalities, and, most importantly, the presence of an abnormal immature T-cell population in pleural fluid.

The hematologic findings deserve particular attention. The initial white blood cell count was normal, and the later leukocytosis was accompanied by neutrophils, lymphocytes, and immature granulocytes rather than reported circulating blasts. Therefore, the leukocytosis itself should not be interpreted as diagnostic evidence of leukemia. The important lesson is that the absence of peripheral blasts does not exclude an underlying lymphoblastic malignancy. In this patient, the diagnosis became apparent through pleural-fluid flow cytometry and was subsequently confirmed by bone marrow examination.

The imaging findings also require careful interpretation. T-cell lymphoblastic malignancies frequently involve the mediastinum, but this patient did not have a clearly defined bulky anterior mediastinal mass. Instead, computed tomography (CT) demonstrated prominent thymic tissue extending anteriorly toward the heart together with bilateral axillary lymphadenopathy and a large localized pleural collection. Therefore, the imaging should not retrospectively be described as demonstrating a definite mediastinal lymphoma mass. The absence of a classic mediastinal mass should not exclude a T-lineage lymphoblastic malignancy.

The pleural-fluid findings were particularly informative. The fluid contained a marked mononuclear predominance, with approximately 99.8% mononuclear cells. Flow cytometry demonstrated that approximately 99% of analyzed cells represented an abnormal immature T-lineage population expressing cytoplasmic CD3 and TdT together with CD1a, CD2, CD4, CD5, CD7, and other T-lineage markers. Flow cytometry is an important tool in the classification of acute leukemia and lymphoblastic neoplasms because immunophenotyping can establish lineage and characterize immature lymphoid populations [7].

Pleural-fluid flow cytometry may therefore be useful when recurrent pleural effusion is unexplained and lymphoma or leukemia is included in the differential diagnosis. Pediatric reports have demonstrated that malignant pleural effusion can occasionally be the presenting manifestation of an underlying hematologic malignancy [1,2]. Other reports have described pleural involvement in T-LBL, sometimes requiring tissue biopsy and immunophenotyping for definitive diagnosis [8,9].

The pleural biopsy in the present case provided additional morphological evidence. The specimen showed fibro-fatty and skeletal muscle tissues heavily infiltrated by atypical small-to-medium-sized lymphoid cells, with numerous macrophages. The pathological conclusion described a morphology resembling non-Hodgkin lymphoma. This finding was important because it demonstrated actual pleural tissue involvement rather than relying exclusively on fluid cytology or flow cytometry.

The subsequent bone marrow examination changed the precise classification of the disease. The marrow was approximately 100% cellular and diffusely infiltrated by blasts, and flow cytometry demonstrated a T-lineage immature phenotype. This established T-ALL rather than T-LBL. T-ALL and T-LBL belong to the same biological spectrum, but the distinction is based largely on the extent of bone marrow involvement; current classification systems recognize this relationship while maintaining the leukemia/lymphoma distinction for clinical purposes [3,4].

An important limitation of this case is the timing of corticosteroid therapy. Methylprednisolone was initiated because of the strong suspicion of an underlying lymphoproliferative malignancy and concern for tumor lysis during the diagnostic process. However, corticosteroids can rapidly reduce lymphoblast burden and potentially affect the morphology or diagnostic yield of subsequent tissue specimens. In this patient, the bone marrow and lymph-node biopsies were performed after three days of Solu-Medrol therapy. Despite this potential limitation, the bone marrow remained extensively involved, with approximately 100% cellularity and a diagnostic T-lineage immunophenotype. The timing of steroid administration should nevertheless be recognized when interpreting the subsequent tissue results.

The infectious differential also required careful consideration. The patient's previous tuberculin skin test was negative, and during the current evaluation, pleural-fluid PCR and acid-fast bacilli testing for tuberculosis were negative. These findings reduced the likelihood of pleural tuberculosis but did not, in isolation, absolutely exclude it. Molecular testing of pleural fluid for tuberculosis has high specificity but variable and often limited sensitivity, meaning that a negative result should be interpreted together with clinical, microbiological, radiological, and histopathological findings [10,11]. In the present case, the absence of fever, normal inflammatory markers, recurrent large-volume effusion, lymphadenopathy, pleural biopsy findings, and subsequent demonstration of T-cell lymphoblastic disease provided a coherent alternative explanation.

The diagnostic pathway in this patient illustrates the value of integrating pleural-fluid analysis, imaging, tissue pathology, flow cytometry, and bone marrow evaluation rather than relying on any single investigation. When an unexplained pleural effusion repeatedly recurs, the differential diagnosis should be broadened rather than repeatedly treating the effusion as an infectious process without establishing its cause.

Another important practical consideration is that pleural fluid should be evaluated comprehensively when malignancy is suspected. Depending on the clinical context, this includes cell count and differential, protein, LDH, glucose, pH, Gram stain and bacterial culture, appropriate tuberculosis studies, cytological examination, and flow cytometry when an abnormal lymphoid population is suspected. Early communication between the clinical, pathology, hematology, and laboratory teams can facilitate appropriate specimen handling and prevent loss of diagnostic material.

The current case also differs from a typical presentation of T-LBL because the eventual diagnosis was T-ALL with extensive marrow involvement. The pleural disease was clinically prominent at presentation, while the hematologic diagnosis became evident through subsequent marrow evaluation. This emphasizes that the presenting anatomical site does not necessarily determine the final hematologic classification.

Published pediatric experience demonstrates that T-lineage lymphoblastic malignancies can present with significant thoracic and pleural disease. T-LBL characteristically involves the mediastinum and may be associated with pleural or pericardial effusions, whereas extensive pleural involvement has also been reported in children with T-LBL [5,8]. In addition, malignant pleural effusion has been reported as an unusual presentation of acute leukemia in children without an obvious mediastinal mass [2]. This case therefore contributes an additional diagnostic perspective: recurrent pleural effusion may precede recognition of T-ALL, and the absence of circulating blasts or a classic mediastinal mass should not lead to premature exclusion of a lymphoblastic malignancy.

At the time of manuscript preparation, the patient had not yet started disease-specific chemotherapy. Therefore, treatment response, minimal residual disease, relapse status, and long-term outcome cannot appropriately be reported. Similarly, no molecular prognostic conclusions are drawn because molecular testing and molecularly directed treatment decisions were not part of the available case data. The report is consequently focused on the diagnostic process and the importance of early recognition.

## Conclusions

Recurrent pleural effusion in a child should prompt a broad diagnostic evaluation when the cause remains unexplained, particularly when the effusion is large, rapidly recurrent, or associated with constitutional symptoms, lymphadenopathy, or hematologic abnormalities. This case demonstrates that T-ALL can initially present predominantly through recurrent pleural disease, even without reported peripheral blood blasts and without a clearly defined bulky anterior mediastinal mass.

Pleural-fluid flow cytometry was a pivotal component of the diagnostic pathway, identifying an abnormal immature T-lineage population that led to further tissue and bone marrow evaluation. Pleural biopsy demonstrated lymphoid infiltration, while subsequent bone marrow examination established extensive T-lineage leukemic involvement and confirmed the diagnosis of T-ALL. The case also emphasizes the importance of distinguishing T-ALL from T-LBL according to the extent of bone marrow involvement. The patient's 100% marrow involvement supported classification as T-ALL despite the prominent pleural and lymph-node manifestations. Finally, recurrent drainage alone should not substitute for establishing the underlying cause of a persistent pleural effusion. Early integration of pleural-fluid studies, flow cytometry, imaging, tissue pathology, and bone marrow examination can substantially shorten the diagnostic pathway and facilitate timely referral to pediatric hematology-oncology.
